# Supplementary material for: Distinctive phenotypes and functions of innate lymphoid cells in human decidua during early pregnancy
Source: Nat Commun. 2020 Jan 20;11:381. doi: 10.1038/s41467-019-14123-z (PMC6971012; doi:10.1038/s41467-019-14123-z)

Supplementary Information

Distinctive Phenotypes and Functions of Innate Lymphoid cells in Human Decidua during early pregnancy

Huhn, Ivarsson, et al

- 10 Supplementary Figures
- Supplementary Figure Legends
- 5 Supplementary Tables

Supplementary Figure Legends

Supplementary Figure 1. Validation of mass cytometry by flow cytometry.

(A) Cryopreserved PBMCs and Decidual Mononuclear Cells (dMCs) were stained for indicated markers (table S2) by mass cytometry and flow cytometry. NK cells = Lin- CD56+, T cells = CD3+ CD19- CD14- CD56-. (B) Correlation of marker expression by CyTOF and FACS (n=3 donors). Two-tailed p-value calculated for Pearson correlation coefficients.

Supplementary Figure 2. Effects of cryopreservation

(A) Matched stains of cryopreserved vs freshly isolated Decidual Mononuclear Cells (dMCs) by CyTOF for indicated markers (Table S3). dNK cells = Lin- CD56+, dT cells = CD3+ CD19- CD14- CD56-, dB cells = CD19+CD3-CD14-CD56-. (B) Correlation of selected markers stained in cryopreserved vs freshly isolated DMCs by CyTOF and FACS (n=9). Two-tailed p-value calculated for Pearson correlation coefficients. (C) Example CD49a staining of Lin-CD56+ dMCs by CyTOF. Stains from cryopreserved and freshly isolated DMCs are shown for 2 donors. (D) Representative 2D plot of CD56 vs CD49a. Cryopreserved Lin- CD56+ dILC in pink are overlaid by Cluster 4 in blue. (E) Radar plot of Cluster 4 response to 4 hour PMA/ionomycin stimulation. Values represent stimulation minus unstimulated media control (n=12). (F) Donut plot showing the average frequencies of identified clusters from fresh tissue preparations (n =7) for comparison with results from cryopreserved cells as shown in Figure 2C (cluster 4 excluded from both).

Supplementary Figure 3. Gating strategy to Lin- cells by CyTOF

Gating strategy reads as follows: Beads excluded, Singlets, Live, CD45+, CD3-CD14-CD19-HLA-DR-

Supplementary Figure 4. Marker expression on Fig. 1 tSNE landscape of matched blood and decidua Lineage negative cells

tSNE landscape was generated in Figure 1 using matched blood and decidua derived Lin- cells (n=6). tSNE are coloured by indicated marker expression. Red=high expression, Blue=low expression.

Supplementary Figure 5. Comparison of scRNA-seq dataset (Vento-Tormo 2018) vs CyTOF for selected ILC subsets

Violin plots show the RNA profiles of dNK1-3, dNKp and dILC3 from Vento-Tormo 2018, compared with indicated CyTOF marker expression levels for analogous Lin- CD56+ densVM clusters identified in Figure 2 (n=12).

Supplementary Figure 6. CD103 vs NKp44 gating in tSNE landscape

(A) Representative 2D CyTOF plot of CD103 vs NKp44 staining of Decidual Lin-CD56+CD127-CD117- cells (B) Regions of tSNE-space occupied by indicated subsets (n=12). This is the same tSNE map generated in Fig. 2. (C) Fig. 2A (Shown for reference). (D) Overlay of subsets from fig. S6B (n=12). Orange = CD103- NKp44+, Red = CD103+ NKp44+, Blue = CD103+ NKp44-, Green = CD103- NKp44-.

Supplementary Figure 7. Response of decidual ILC subsets to stimulation by PMA/ionomycin

Decidual mononuclear cells were stimulated with PMA plus ionomycin for 4 hours in the presence of protein transport inhibitors and analysed as shown in Fig. 3A. A new tSNE landscape was generated using the markers indicated in the final column of table S1. Lin-CD56+ subsets identified in Fig. 2 were manually gated in the tSNE plot. Scatter plots show proportion of cells within each subset staining for functional readouts or Ki-67 (n=12). Values represent stimulation minus unstimulated media control for each subset. Error bars represent SD. Plots show the characteristic pattern of responses of each ILC subset for the seven different functional readouts upon stimulation.

Supplementary Figure 8. Response of decidual ILC to stimulation by 'missing self'

(A) Decidual mononuclear cells were co-cultured with K562 for 6 hours in the presence of protein transport inhibitors. Scatter plots show functional output and Ki-67 staining for Lin-CD56+ subsets (n= 8-11). Values represent stimulation minus unstimulated media control for each subset. (B) Data is summarised in the scatter plots by subset (n=8). In A) Two-tailed One-Way ANOVA of matched data points with Tukey's correction were performed. P values of comparisons for A) are found in table S5. Error bars represent SD.

Supplementary Figure 9. KIR-co-expression and forward or side scatter analysed by flow cytometry

(A) Forward scatter of Lin- CD56+ Decidual Mononuclear cells (dMCs) does not increase with increasing number of co-expressed KIRs (n=11). (B) Side-scatter in dNK cell subsets co-expressing only inhibitory KIRs (left, n=9) or only activating KIRs (right, n =4). Stars indicate significance obtained with Two-tailed One-Way ANOVA of matched data points with Tukey correction. ** = $p < 0.01$, *** = $p < 0.001$

Supplementary Figure 10. Decidual NK cells contain distinct amounts of cytotoxic molecules compared to pbNK

(A) CD14-CD19-CD3- lymphocytes are gated for decidual NK (dNK), as well as $CD56^{bright}$ and $CD56^{dim}$ pbNK cells in PBMC, respectively. (B) Histograms indicate content of perforin, Granzyme A, B and Granulysin (9/15 kDa) in dNK and in the indicated subsets of pbNK. Bottom line of graphs show aggregated data for dNK and $pbNK^{bright}$ and $pbNK^{dim}$ (n=5-13), color coded as in B top row. Red Line represents mean. A two-tailed Mann-Whitney test was performed for Perforin and Granzyme A. Unpaired t-tests were used for Granzyme B and Granulysin. * = $p < 0.05$, ** = $p < 0.01$, *** = $p < 0.001$

Supplementary Tables

Supplementary Table 1. CyTOF Antibody list

Isotope	Isotope Source	Marker	Antibody Clone	Antibody Source	viSNE Generation*
89Y	Fluidigm Sciences	CD45	HI30	Fluidigm Sciences	
112Cd		CD3 Qdot605	UCHT1	Thermofisher	
112Cd		CD14 Qdot605	Tük4	Thermofisher	
112Cd		CD19 Qdot605	SJ25-C1	Thermofisher	
112Cd		HLA-DR Qdot605	Tü36	Thermofisher	
115In	Sigma	CD57	HCD57	BioLegend	x
141Pr	Fluidigm Sciences	KIR2DS4	FES172	Beckman Coulter	x
142Nd	Fluidigm Sciences	CD103	Ber-ACT8	BioLegend	x
143Nd	Fluidigm Sciences	CD117	104D2	Fluidigm Sciences	x
144Nd	Fluidigm Sciences	CD69	FN50	Fluidigm Sciences	x
146Nd	Fluidigm Sciences	Granzyme B	CLB-GB11	Novus	x
147Sm	Fluidigm Sciences	MIP1β	D21-1351	BioLegend	
148Nd	Fluidigm Sciences	NKp30	P30-15	BioLegend	x
149Sm	Fluidigm Sciences	KIR2DL2/L3/S2	GL183	Beckman Coulter	x
150Nd	Fluidigm Sciences	IL-22	22URTI	Fluidigm Sciences	
151Eu	Fluidigm Sciences	CD107a	H4A3	Fluidigm Sciences	
152Sm	Fluidigm Sciences	Eomes	WD1928	eBioscience	x
153Eu	Fluidigm Sciences	MIP1α	1.2_3E8-2H6-2B6	Peprotech	
154Sm	Fluidigm Sciences	CD96	NK92.39	BioLegend	x
155Gd	Fluidigm Sciences	CD56	B159	Fluidigm Sciences	x
156Gd	Fluidigm Sciences	LILRB1	GHI/75	Fluidigm Sciences	x
157Gd	Trace Sciences	NKG2C	134591	R&D Systems	x
158Gd	Fluidigm Sciences	IFN-γ	B27	Fluidigm Sciences	
159Tb	Fluidigm Sciences	GM-CSF	BVD2-21C11	Fluidigm Sciences	
160Gd	Fluidigm Sciences	NKp44	P44-8	BioLegend	x
161Dy	Fluidigm Sciences	Tbet	4B10	Fluidigm Sciences	x
162Dy	Fluidigm Sciences	NKp46	BAB281	Fluidigm Sciences	x
163Dy	Fluidigm Sciences	CD49a	TS2/7	Fluidigm Sciences	x
164Dy	Fluidigm Sciences	CD161	HP-3G10	Fluidigm Sciences	x
165Ho	Fluidigm Sciences	CD127	A019D5	Fluidigm Sciences	x
166Er	Fluidigm Sciences	NKG2D	ON72	Fluidigm Sciences	x
167Er	Fluidigm Sciences	KIR3DL1	DX9	Fluidigm Sciences	x
169Tm	Fluidigm Sciences	NKG2A	Z199	Fluidigm Sciences	x
170Er	Fluidigm Sciences	XCL1	109001	R&D Systems	
171Yb	Fluidigm Sciences	DNAM-1	DX11	Fluidigm Sciences	x
172Yb	Fluidigm Sciences	Ki-67	B56	Fluidigm Sciences	x
173Yb	Fluidigm Sciences	KIR2DL1	143211	R&D Systems	x

174Yb	Fluidigm Sciences	CD94	HP-3D9	Fluidigm Sciences	x
175Lu	Fluidigm Sciences	AhR	FF3399	eBioscience	x
176Yb	Fluidigm Sciences	KIR2DL3	180701	R&D Systems	x
209Bi	Fluidigm Sciences	CD16	3G8	Fluidigm Sciences	x

* ‘X’ denotes markers that were used to generate tSNE landscapes where indicated in the text

Supplementary Table 2. Selected Antibodies used in mass vs flow cytometry comparison

Marker	Clone	Isotope	Clone	Fluorophore
KIR2DL1	143211	166Er	REA284	APC-Vio770
KIR2DL2/S2/L3	GL183	149Sm	GL183	PerCP-Cy5.5
KIR3DL1	DX9	163Er	DX9	BV421
CD56	B159	174Yb	HCD56	PE-Dazzle
CD16	3G8	209Bi	3G8	Bv650
NKG2A	Z199	171Yb	REA110	APC
CD19	SJ25-C1	142Nd	HIB19	AF700
CD3	UCHT1	Er170	Okt3	Bv510
CD4	SK3	145Nd	Okt4	PE-Cy5
CD8	(RPA-T8)	146Nd	SK1	FITC

Supplementary Table 3. Antibodies used in fresh vs cryopreserved comparison

Marker	Clone	Isotope	Clone	Fluorophore
KIR2DL1	143211	166Er	REA284	APC-Vio770
KIR2DL2/S2/L3	GL183	149Sm	GL183	PerCP-Cy5.5
KIR3DL1/S1	Z27	151Eu		
KIR2DS4	FES172	141Pr	JJC11.6	APC
CD56	NCAM16.2	174Yb	HCD56	PE-Dazzle
CD16	3G8	148Nd	3G8	Bv650
NKG2A	Z199	171Yb	REA110	APC
CD19	SJ25-C1	142Nd	HIB19	AF700
CD3	UCHT1	170Er	Okt3	BV510
CD4	SK3	145Nd	Okt4	PE-Cy5
CD8	(RPA-T8)	146Nd	SK1	FITC
2B4	2-69	143Nd		
CD7	CD7-6B7	147Sm		
CD9	SN4	159Tb		
CD69	FN50	162Dy		
CD57	HCD57	115In		
CD27	L128	167Er		
CD122	TU27	144Nd		
CD94	DX22	165Ho		
NKp44	P44-8	160Gd		
NKp46	195314	155Gd		
TCRVD2	B6	152Sm		
KIR3DL1	DX9	163Dy		

Supplementary Table 4. P-values for PMA stimulation assays of dILC

Comparisons	CD107a	GM-CSF	IFN-γ	Ki-67	MIP1α	MIP1β	XCL1
dNK1 vs. dNK2	0.001	0.0005	0.0244	0.0017	0.0089	0.0031	0.0005
dNK1 vs. dNK3	< 0.0001	0.0002	0.0038	0.0033	0.0013	0.0036	0.0007
dNK1 vs. dNKp	0.0005	0.0048	0.1957	0.0002	0.0098	0.0009	0.0034
dNK1 vs. dILC3	0.1348	0.3046	> 0.9999	0.0038	0.0645	0.004	0.9702
dNK1 vs. pb-like	0.0481	> 0.9999	0.0498	0.0021	0.9974	0.9922	0.9973
dNK2 vs. dNK3	0.0003	0.0504	0.0149	0.9891	0.7233	0.3647	0.331
dNK2 vs. dNKp	< 0.0001	0.0001	0.037	0.0001	0.0018	< 0.0001	< 0.0001
dNK2 vs. dILC3	0.0043	0.0036	0.0454	0.2571	0.0055	< 0.0001	0.0003
dNK2 vs. pb-like	0.0177	0.0009	0.677	0.0203	0.0011	0.0053	0.0032
dNK3 vs. dNKp	< 0.0001	< 0.0001	0.0052	0.0001	0.0003	< 0.0001	< 0.0001
dNK3 vs. dILC3	0.0002	0.0016	0.0044	0.0943	0.0013	< 0.0001	0.0001
dNK3 vs. pb-like	0.0002	0.0003	0.5641	0.1004	0.0002	0.0006	0.0004
dNKp vs. dILC3	0.9438	< 0.0001	0.3757	< 0.0001	0.3213	0.4419	0.0591
dNKp vs. pb-like	0.0008	0.2225	0.0551	0.0001	0.0418	0.0249	0.2362
dILC3 vs. pb-like	0.0546	0.7047	0.0886	0.9671	0.256	0.0317	> 0.9999

Two-tailed One-Way ANOVA of matched data points with Tukey's correction were performed

Supplementary Table 5. P-values for 'missing self' stimulation assays of dILC

Comparisons	CD107a	GM-CSF	IFN-γ	Ki- 67	MIP1α	MIP1β	XCL1
dNK1 vs. dNK2	0.0037	0.0015	0.0127	0.0034	0.0083	< 0.0001	< 0.0001
dNK1 vs. dNK3	0.0293	0.3862	0.012	0.0445	0.0465	0.0061	0.004
dNK1 vs. dNKp	0.0002	< 0.0001	0.0008	< 0.0001	0.0069	< 0.0001	0.0001
dNK1 vs. dILC3	0.0056	0.9634	0.0327	0.0189	0.2145	< 0.0001	0.0095
dNK1 vs. pb-like	0.0279	0.1531	0.018	0.0011	0.9832	0.5911	0.5223
dNK2 vs. dNK3	0.8357	0.0049	0.999	0.0684	0.3384	0.0157	0.0068
dNK2 vs. dNKp	0.0001	0.0001	0.001	< 0.0001	0.0014	< 0.0001	< 0.0001
dNK2 vs. dILC3	0.0008	0.0182	0.0033	0.8345	0.0087	< 0.0001	< 0.0001
dNK2 vs. pb-like	0.0877	0.0006	0.8148	0.1085	0.1697	0.036	< 0.0001
dNK3 vs. dNKp	0.0001	< 0.0001	0.0016	< 0.0001	0.0015	< 0.0001	< 0.0001
dNK3 vs. dILC3	0.0019	0.6416	0.0071	0.9603	0.0667	< 0.0001	< 0.0001
dNK3 vs. pb-like	0.5838	0.0095	0.6081	0.0107	0.8851	0.4944	< 0.0001
dNKp vs. dILC3	0.9208	0.2301	0.8849	< 0.0001	0.9997	> 0.9999	0.1244
dNKp vs. pb-like	0.0001	0.0055	0.0013	< 0.0001	0.2461	0.001	0.0075
dILC3 vs. pb-like	< 0.0001	0.9987	0.0015	0.2431	0.3585	0.0004	0.0688

Two-tailed One-Way ANOVA of matched data points with Tukey's correction were performed

Supplementary Figure 1 (Huhn and Ivarsson et al.)

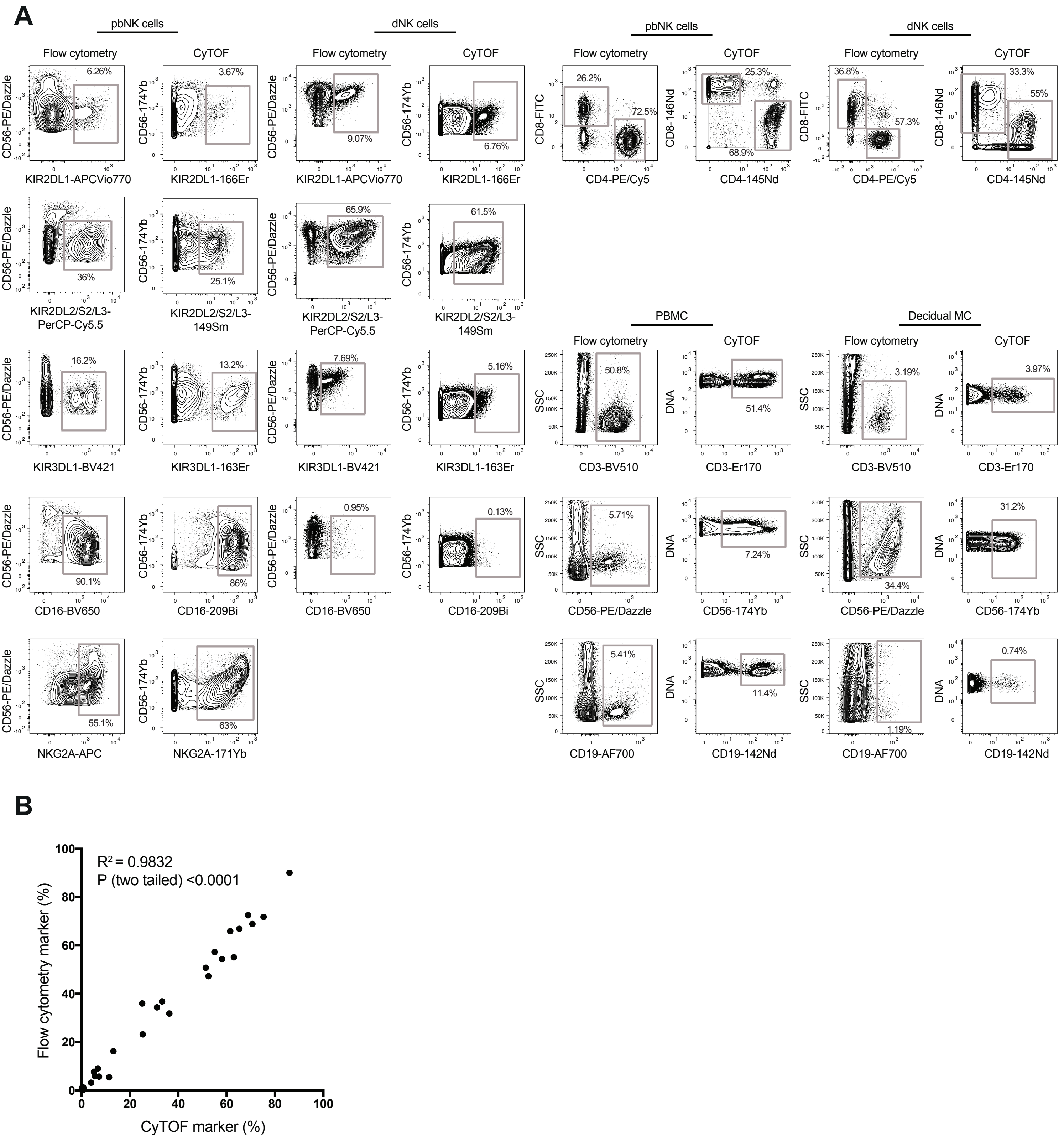

Supplementary Figure 2 (Huhn and Ivarsson et al.)

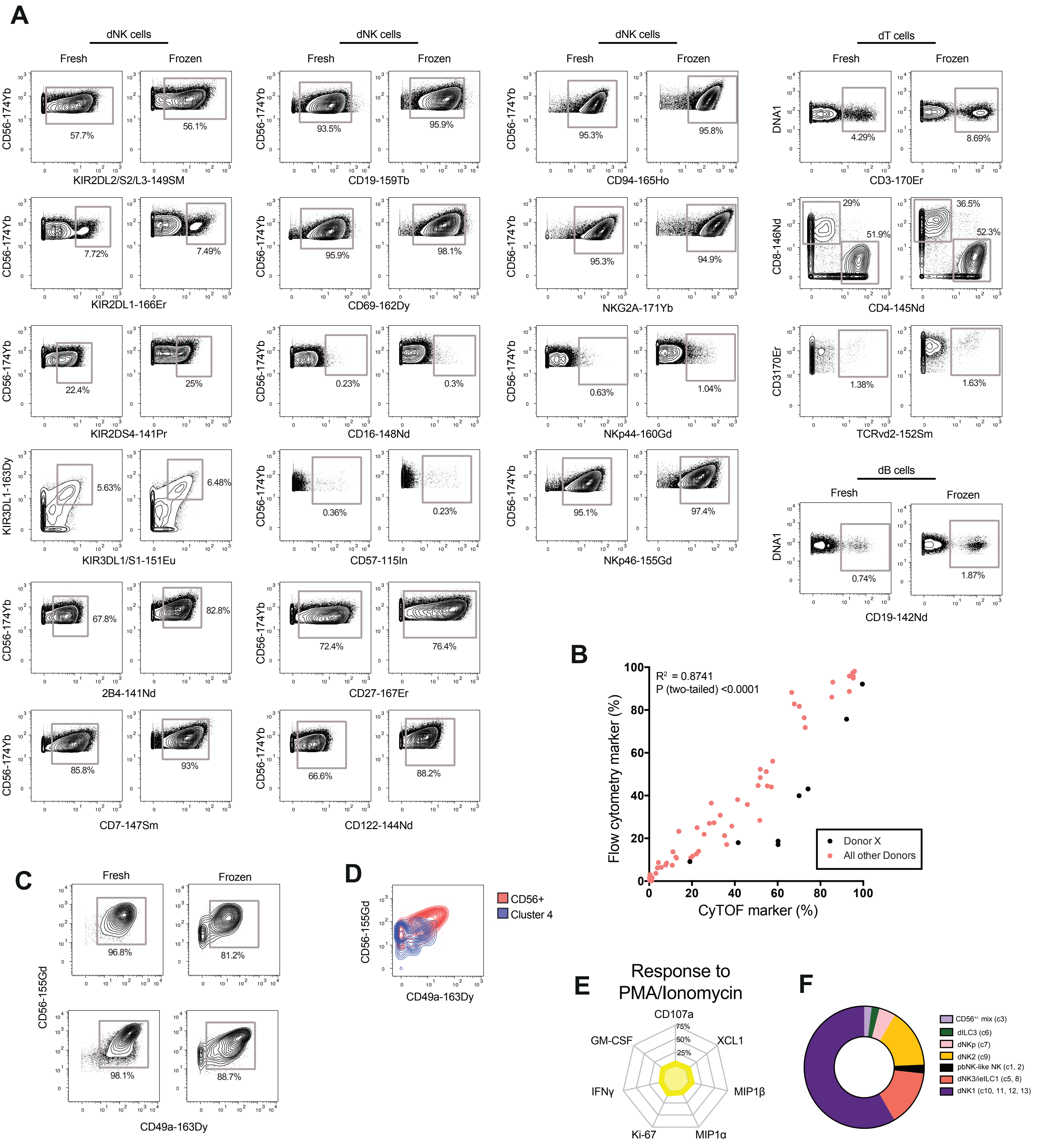

Supplementary Figure 3 (Huhn and Ivarsson et al.)

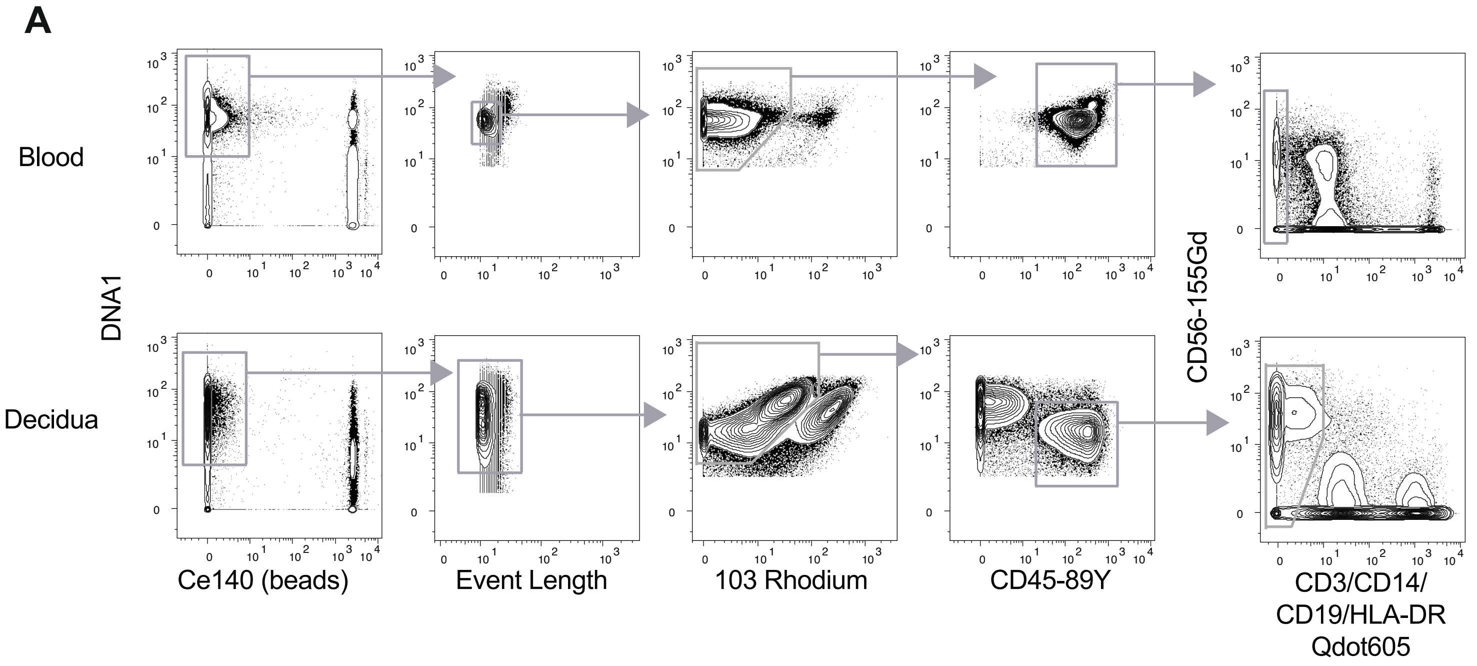

Supplementary Figure 4 (Huhn and Ivarsson et al.)

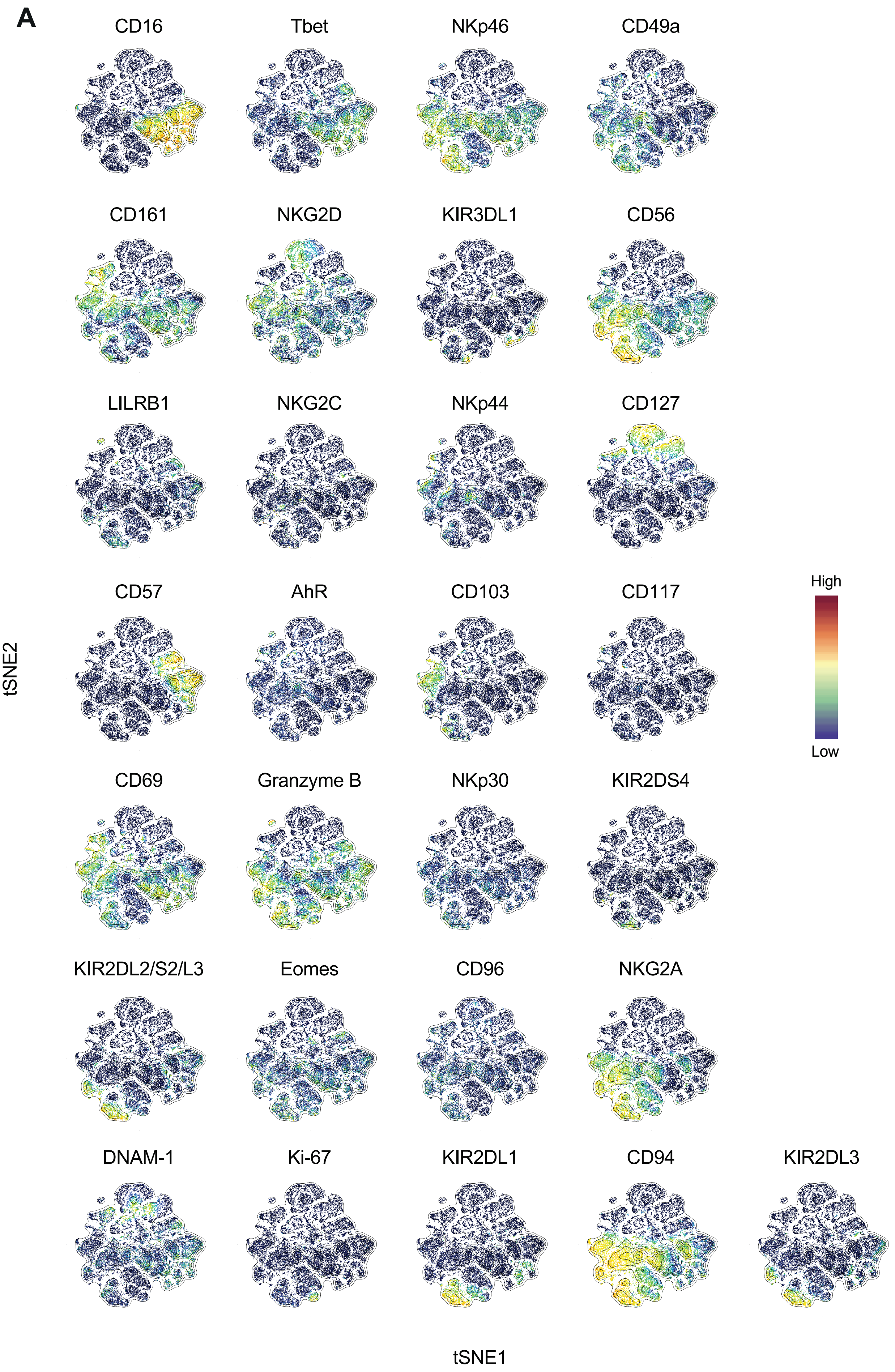

Supplementary Figure 5a (Huhn and Ivarsson et al.)

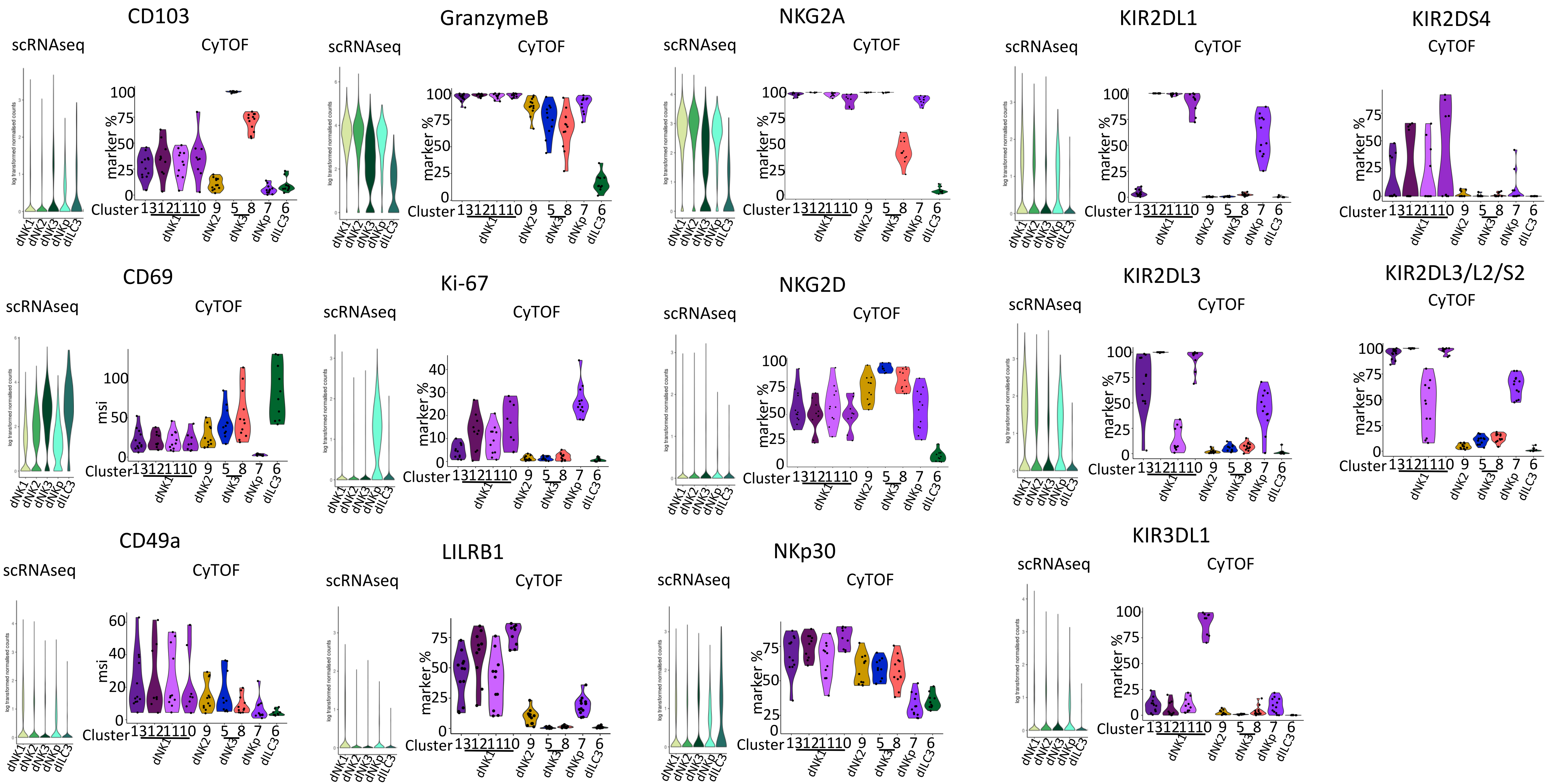

Supplementary Figure 5b (Huhn and Ivarsson et al.)

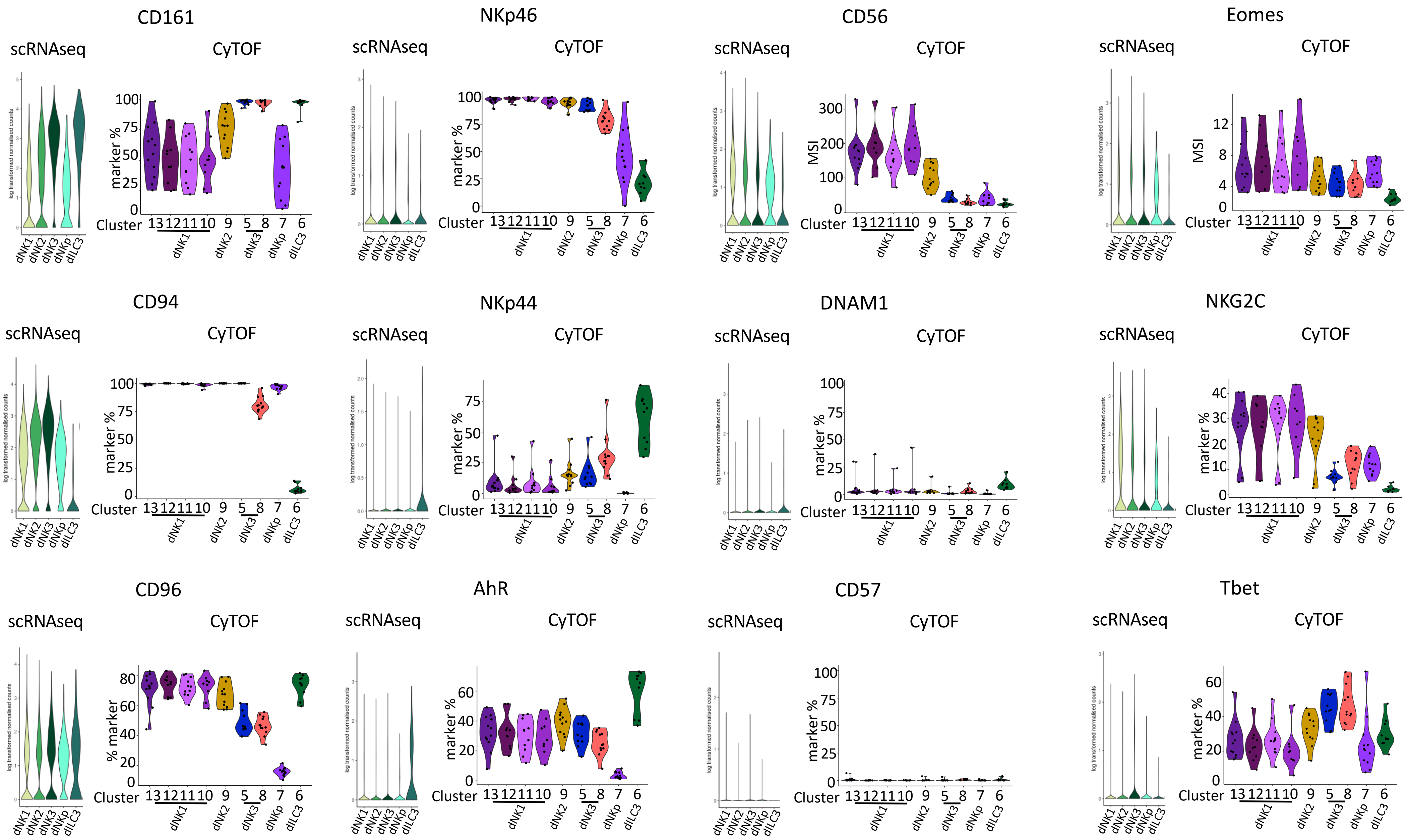

Supplementary Figure 6 (Huhn and Ivarsson et al.)

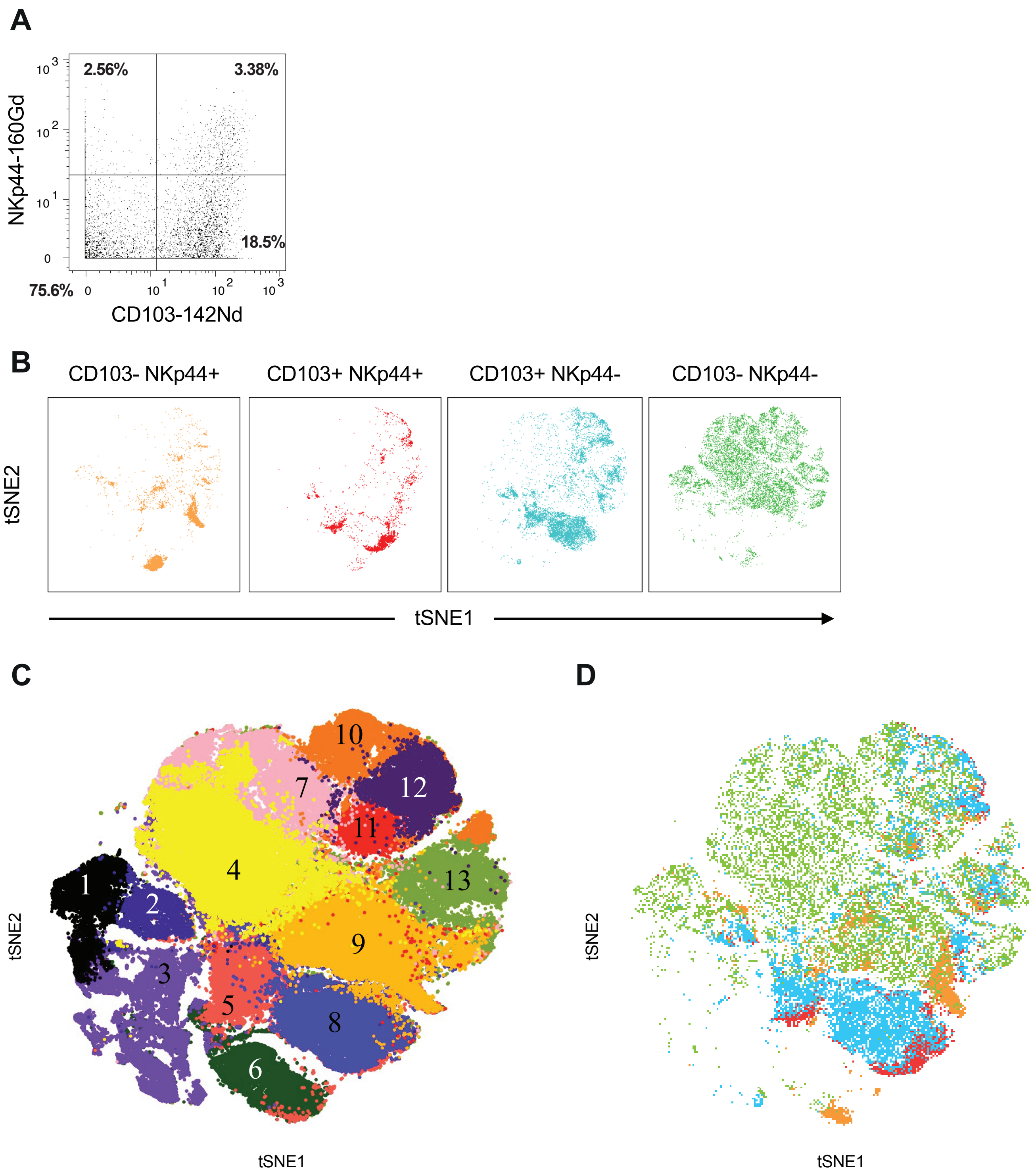

Supplementary Figure 7 (Huhn and Ivarsson et al.)

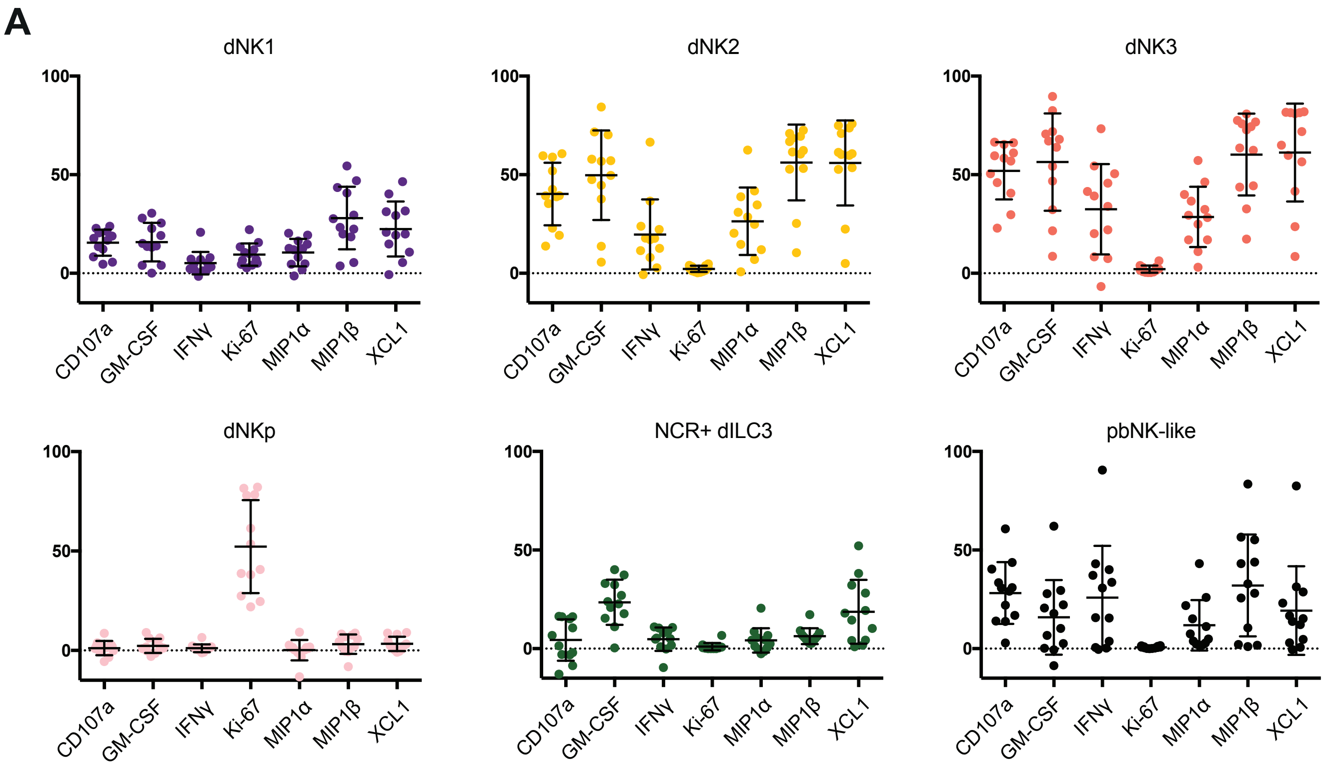

Supplementary Figure 8 (Huhn and Ivarsson et al.)

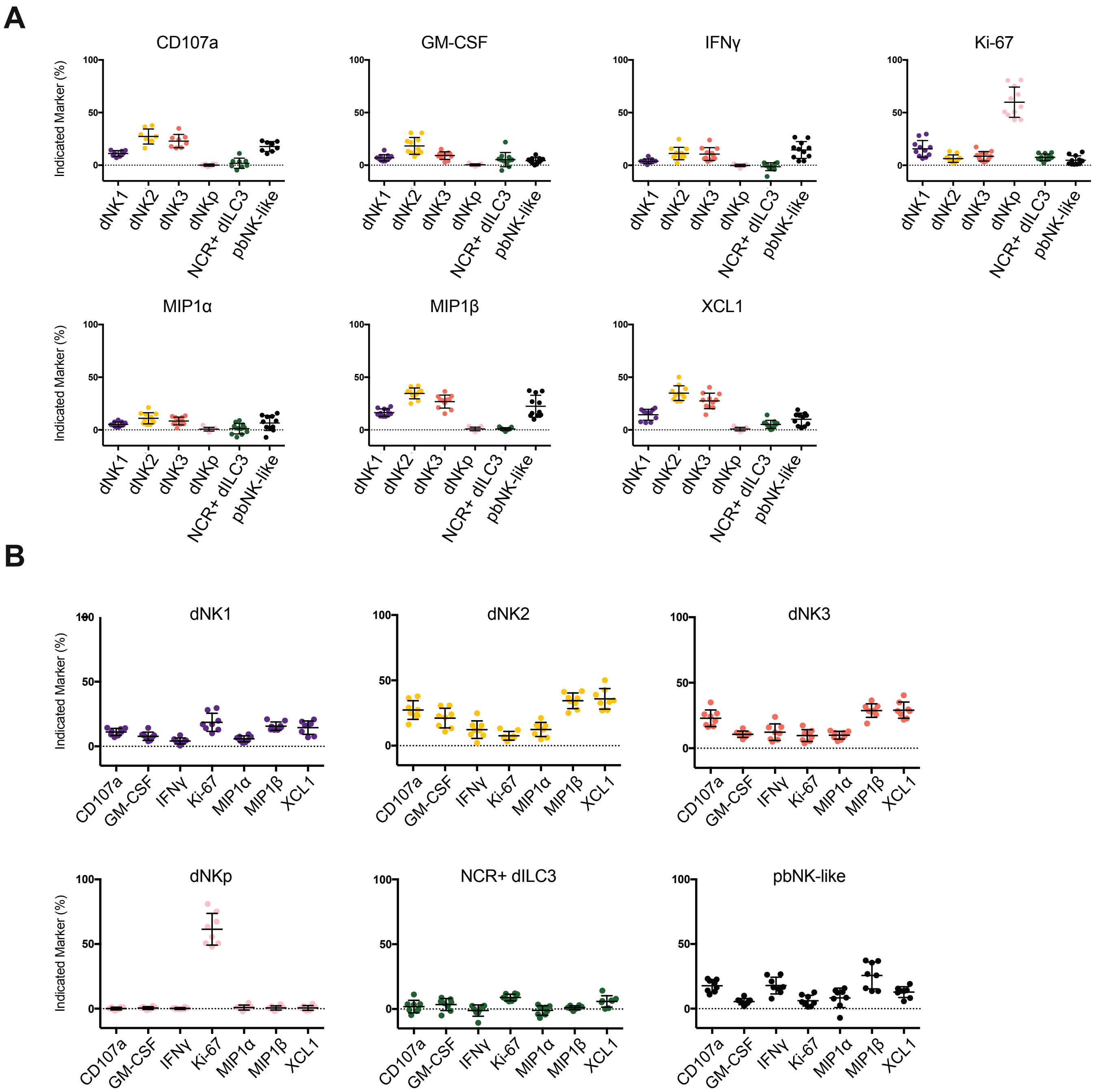

Supplementary Figure 9 (Huhn and Ivarsson et al.)

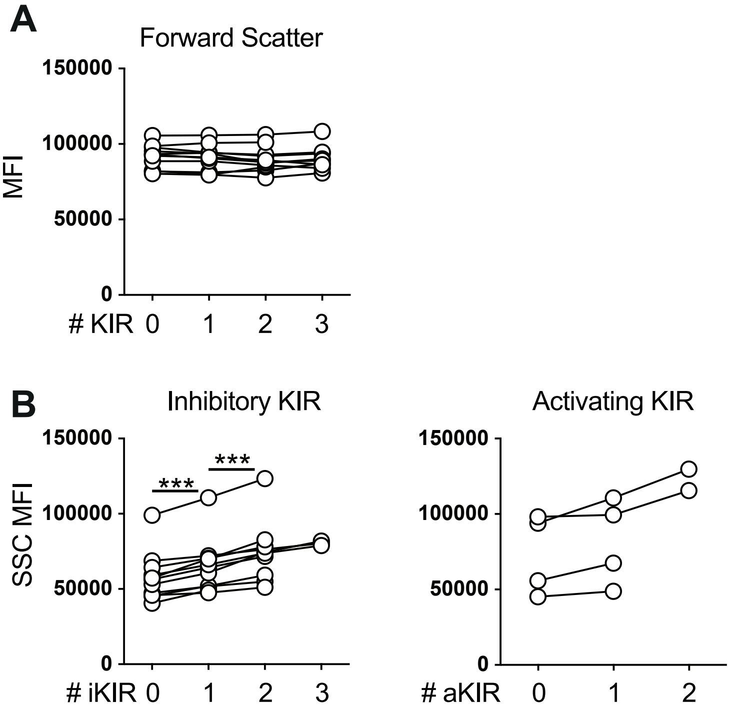

Supplementary Figure 10 (Huhn and Ivarsson et al.)

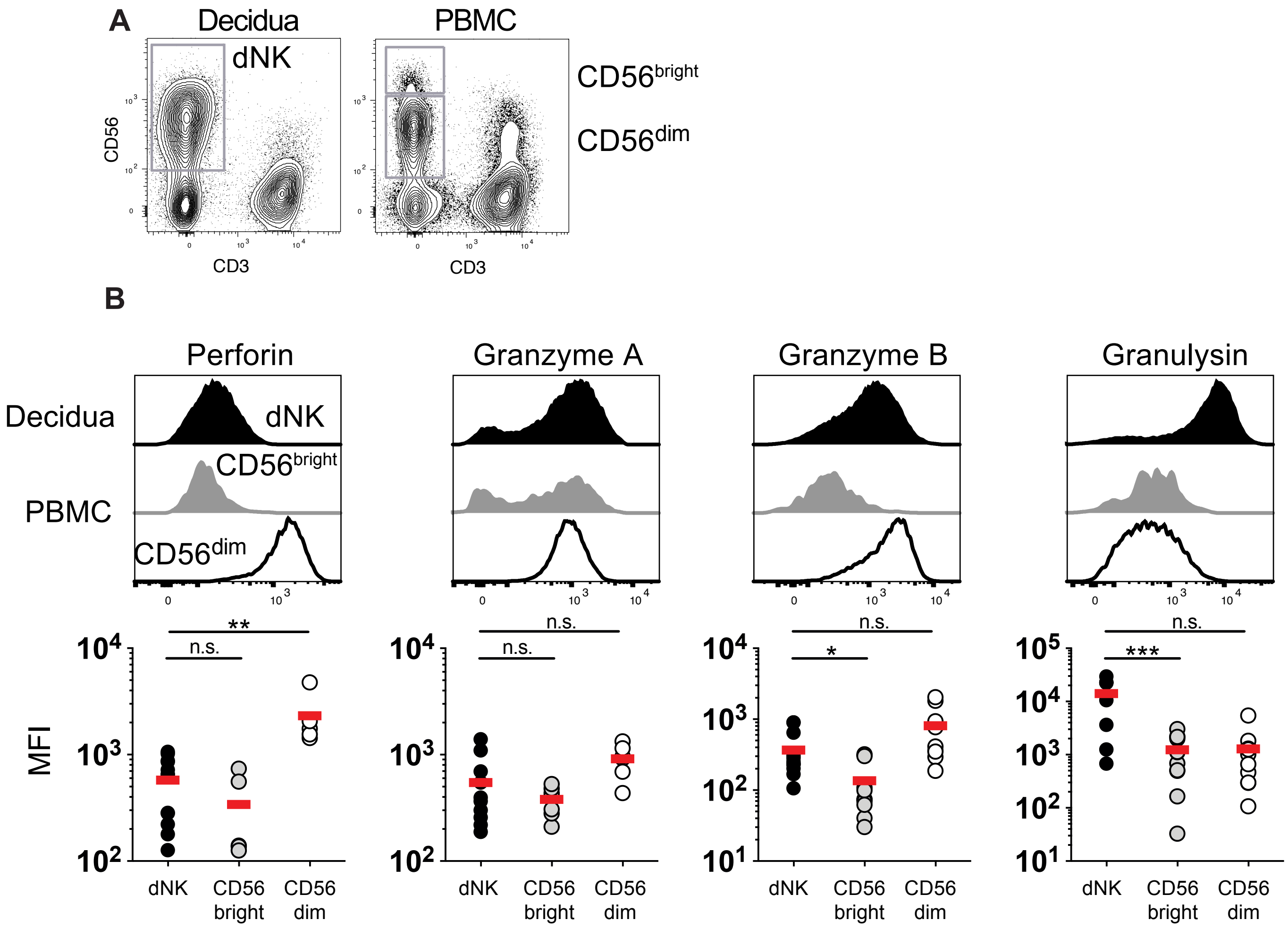

Supplement: Supplementary file 1 — Supplementary Information [file 41467_2019_14123_MOESM1_ESM.pdf]
