# Supplementary material for: Distinctive phenotypes and functions of innate lymphoid cells in human decidua during early pregnancy
Source: Nat Commun. 2020 Jan 20;11:381. doi: 10.1038/s41467-019-14123-z (PMC6971012; doi:10.1038/s41467-019-14123-z)
Supplement: Supplementary file 3 — Reporting Summary [file 41467_2019_14123_MOESM3_ESM.pdf]

nature research

Corresponding author(s): Francesco Colucci

Last updated by author(s): Dec 11, 2019

Reporting Summary

Nature Research wishes to improve the reproducibility of the work that we publish. This form provides structure for consistency and transparency in reporting. For further information on Nature Research policies, see Authors & Referees and the Editorial Policy Checklist.

Statistics

For all statistical analyses, confirm that the following items are present in the figure legend, table legend, main text, or Methods section.

n/a	Confirmed	
☐	☒	The exact sample size (*n*) for each experimental group/condition, given as a discrete number and unit of measurement
☐	☒	A statement on whether measurements were taken from distinct samples or whether the same sample was measured repeatedly
☐	☒	The statistical test(s) used AND whether they are one- or two-sided<br>*Only common tests should be described solely by name; describe more complex techniques in the Methods section.*
☐	☒	A description of all covariates tested
☐	☒	A description of any assumptions or corrections, such as tests of normality and adjustment for multiple comparisons
☐	☒	A full description of the statistical parameters including central tendency (e.g. means) or other basic estimates (e.g. regression coefficient) AND variation (e.g. standard deviation) or associated estimates of uncertainty (e.g. confidence intervals)
☐	☒	For null hypothesis testing, the test statistic (e.g. *F*, *t*, *r*) with confidence intervals, effect sizes, degrees of freedom and *P* value noted<br>*Give P values as exact values whenever suitable.*
☒	☐	For Bayesian analysis, information on the choice of priors and Markov chain Monte Carlo settings
☒	☐	For hierarchical and complex designs, identification of the appropriate level for tests and full reporting of outcomes
☐	☒	Estimates of effect sizes (e.g. Cohen's *d*, Pearson's *r*), indicating how they were calculated

Our web collection on statistics for biologists contains articles on many of the points above.

Software and code

Policy information about availability of computer code

Data collection	Patient material was collected as described in the methods and materials. Sample data was acquired on an Helios (Fluidigm) and BD LSR Fortessa (BD Biosciences)
Data analysis	FCS files were analysed with FlowJo v10.5.3 (Tree Star Inc.). tSNE, DensVM clustering and nearest neighbour-based residual probability analyses were performed using the R packages cytofkit (v.1.6.5) and DepecheR (v1.1.9) from Bioconductor (Chen et al., 2016; Theorell et al., 2019).<br>Statistical analyses were largely performed using PRISM (GraphPad Software Inc.) and the open source statistical package R (www.r-project.org). Datasets were tested for normal distribution and the appropriate statistical test was then used to compare subsets. Methods used are specified in figure legends.

For manuscripts utilizing custom algorithms or software that are central to the research but not yet described in published literature, software must be made available to editors/reviewers. We strongly encourage code deposition in a community repository (e.g. GitHub). See the Nature Research guidelines for submitting code & software for further information.

Data

Policy information about availability of data

All manuscripts must include a data availability statement. This statement should provide the following information, where applicable:

- Accession codes, unique identifiers, or web links for publicly available datasets
- A list of figures that have associated raw data
- A description of any restrictions on data availability

The authors declare that the data supporting the findings of this study are available within the paper and its supplementary information file. The source data underlying Figs 2C, 3A-D, 4A-D, 5A-I, 6A-D, and Supplementary Figs 1B, 2B, 2E-F, 5, 7, 8A-B, 9A-B, 10B are provided as Source Data file. Data not found in the source data are available upon request from the authors.

Field-specific reporting

Please select the one below that is the best fit for your research. If you are not sure, read the appropriate sections before making your selection.

☒ Life sciences ☐ Behavioural & social sciences ☐ Ecological, evolutionary & environmental sciences

For a reference copy of the document with all sections, see nature.com/documents/nr-reporting-summary-flat.pdf

Life sciences study design

All studies must disclose on these points even when the disclosure is negative.

Sample size	Up to 19 donors were used for a figure (In the source data file). This number was dictated by sample availability and to permit statistical calculations. In some instances samples were selected with particular HLA-C genotypes
Data exclusions	No donors were excluded. But for FACS and CyTOF analysis, data points were excluded if fewer than 100 cells fell into a gate
Replication	Data could be reproduced. Stains from fresh and cryopreserved samples were compared to assess the effect of cryopreservation on the data.
Randomization	Donors were not grouped in a particular way. Samples differed by tissue of origin. For stimulation by 'missing self' donors were selected based on HLA-C status.
Blinding	No blinding was performed.

Reporting for specific materials, systems and methods

We require information from authors about some types of materials, experimental systems and methods used in many studies. Here, indicate whether each material, system or method listed is relevant to your study. If you are not sure if a list item applies to your research, read the appropriate section before selecting a response.

Materials & experimental systems

n/a	Involved in the study	
☐	☒	Antibodies
☐	☒	Eukaryotic cell lines
☒	☐	Palaeontology
☒	☐	Animals and other organisms
☐	☒	Human research participants
☒	☐	Clinical data

Methods

n/a	Involved in the study	
☒	☐	ChIP-seq
☐	☒	Flow cytometry
☒	☐	MRI-based neuroimaging

Antibodies

Antibodies used

For CyTOF:
89Y-CD45 (HI30) Fluidigm Sciences Cat# 3141009B
CD3 Qdot605 (UCHT1) Thermofisher Cat# Q10054
CD14 Qdot605 (Tük4) Thermofisher Cat# Q10013
CD19 Qdot605 (SJ25-C1) Thermofisher Cat# Q10306
HLA-DR Qdot605 (Tü36) Thermofisher Cat# Q10052
115In CD57 (HCD57) BioLegend Cat# 322325
141Pr KIR2DS4 (FES172) Beckman Coulter Cat# B33084
142Nd CD103 (Ber-ACT8) BioLegend Cat# 350202
143Nd-CD117 (104D2) Fluidigm Sciences Cat# 3143001B
144Nd- CD69 (FN50) Fluidigm Sciences Cat# 3144018B
146Nd Granzyme B (CLB-GB11) Novus Cat# NBP1-50071
147Sm MIP1b (D21-1351) BioLegend
148Nd NKp30 (P30-15) BioLegend Cat# 325204
149Sm KIR2DL2/L3/S2 (GL183) Beckman Coulter Cat# IM1846
150Nd-IL-22 (22URTI) Fluidigm Sciences Cat# 3150007B
151Eu-CD107a (H4A3) Fluidigm Sciences Cat# 3151002B
152Sm Eomes (WD1928) eBioscience Cat# 14-4877-82
153Eu MIP1a (1.2_3E8-2H6-2B6) Peprotech Cat# 500-M74
154SmCD96 (NK92.39) BioLegend Cat# 338404
155Gd-CD56 (B159) Fluidigm Sciences Cat# 3155008B
156Gd-LILRB1 (GHI/75) Fluidigm Sciences Cat# 3156020B
157Gd NKG2C (134591) R&D Systems Cat# MAB138
158Gd-IFNg (B27) Fluidigm Sciences Cat# 3158017B

159Tb-GM-CSF (BVD2-21C11) Fluidigm Sciences Cat# 3159008B
160Gd NKp44 (P44-8) BioLegend Cat# 325102
161Dy-Tbet (4B10) Fluidigm Sciences Cat# 3161014B
162Dy-NKp46 (BAB281) Fluidigm Sciences Cat# 3162021B
163Dy-CD49a (TS2/7) Fluidigm Sciences Cat# 3163015B
164Dy-CD161 (HP-3G10) Fluidigm Sciences Cat# 3164009B
165Ho-CD127 (A019D5) Fluidigm Sciences Cat# 3165008B
166Er-NKG2D (ON72) Fluidigm Sciences Cat# 3166016B
167Er-KIR3DL1 (DX9) Fluidigm Sciences Cat# 3167013B
169Tm-NKG2A (Z199) Fluidigm Sciences Cat# 3169013B
170Er XCL1 (109001) R&D Systems Cat# mab6951
171Yb-DNAM-1 (DX11) Fluidigm Sciences Cat# 3171013B
172Yb-Ki-67 (B56) Fluidigm Sciences Cat# 3172024B
173Yb KIR2DL1 (143211) R&D Systems Cat# MAB1844
174Yb-CD94 (HP-3D9) Fluidigm Sciences Cat# 3174015B
175Lu AhR (FF3399) eBioscience Cat# 14-9854-82
176Yb KIR2DL3 (180701) R&D Systems Cat# MAB2014
209Bi-CD16 (3G8) Fluidigm Sciences Cat# 3209002B
142Nd-CD19 (SJ25-C1) Fluidigm Sciences Cat# 3142001B
145Nd-CD4 (SK3) Fluidigm Sciences Cat# 3145001B
146Nd-CD8 (RPA-T8) Fluidigm Sciences Cat# 3146001B
147Sm-CD7 (CD7-6B7) Fluidigm Sciences Cat# 3147006B
159Tb CD9 (SN4) Thermofisher Cat# 14-0098-82
162Dy-CD69 (FN50) Fluidigm Sciences Cat# 3162001B
167Er-CD27 (L128) Fluidigm Sciences Cat# 3167006B

Antibodies without catalog no. Custom ordered/conjugated. Contact authors for info.
KIR2DL1 143211 166Er, CD16 3G8 148Nd
KIR3DL1 DX9 163Er, CD3 UCHT1 170Er
CD56 B159 174Yb, 2B4 2-69 143Nd
NKG2A Z199 171Yb, CD57 HCD57 115In
CD122 TU27 144Nd, CD94 DX22 165Ho
KIR3DL1 DX9 163Dy

Flowcytometry Abs:
XCL-1 Unconjugated RnD MAB6951-SP 109001
KIR2DL2/3/S2 PerCP-Cy5.5 Beckman A66900 GL183
NKG2A APC Miltenyi 130-113-563 REA110
GranzymeB AF700 BD 560213 GB11
KIR2DL1 APC Vio770 Miltenyi 130-118-345 REA284
CD107a BV421 Biolegend 328626 H4A3
CD4 BV510 Biolegend 317444 OKT4
CD14 BV510 Biolegend 301842 M5E2
CD19 BV510 Biolegend 302242 HIB19
Viability Aqua ThermoFisher L34957 N/A
KIR2DS4 biotin Miltenyi 130-092-898 JJC11.6
Streptavidin QD605 Thermo Q10001MP N/A
CD56 BV650 Biolegend 318344 HCD56
CCL3/MIP1a PE Miltenyi 130-103-629 REA257
GM-CSF PE-CF594 BD BVD2-21C11 562857
CD3 PE-Cy5 Biolegend 300410 UCHT1
KIR2DL1/S1 PE-Vio770 Miltenyi 130-099-891 11PB6
Perforin BV421 Biolegend 308122 dg9
Granzyme A Alexa Fluor 700 Biolegend 507210 CB9
Granzyme A PE-Cy7 eBioscience/invitrogen 25-9177-42 CB9
Granulysin PE Biolegend 348004 DH2
N/A Atto488 Lightning-Link (Expedeon) SKU: 350-0005 N/A
KIR3DL1 BV421 BioLegend 312714 DX9
CD56-PE-Dazzle (HCD56) BioLegend Cat# 318348
CD16-Bv650 (3G8) BD Biosciences Cat# 563692
CD19-AF700 (HIB19) BD Biosciences Cat# 557921
CD3-Bv510 (OKT3) BioLegend Cat# 317332
CD4-PE-Cy5 (OKT4) BioLegend Cat# 317412
CD8-FITC (SK1) BioLegend Cat# 344704
KIR2DS4-APC (JJC11.6) Miltenyi Cat# 130-099-709

For confocal microscopy:
Perforin Unconjugated Biolegend 308102 dg9
Granzyme A Unconjugated Biolegend 507202 CB9
Granulysin Unconjugated Biolegend 348008 DH2
Pericentrin Unconjugated abcam ab99341 ab99341
Phalloidin Alexa 647 Thermo Fisher A22287 N/A

Validation	All antibodies were titrated, functional markers were titrated on PMA plus ionomycin stimulated cells. Antibodies were validated by the manufacturers. For a subset of markers, CyTOF stains and FACS stains were compared.

Eukaryotic cell lines

Policy information about cell lines

Cell line source(s)	K562 and P815 purchased from DSMZ
Authentication	The cells are commercially available from DSMZ and therefore authenticated
Mycoplasma contamination	Cell lines tested negative for mycoplasma
Commonly misidentified lines (See ICLAC register)	*Name any commonly misidentified cell lines used in the study and provide a rationale for their use.*

Human research participants

Policy information about studies involving human research participants

Population characteristics	These are all women undergoing elective terminations of healthy pregnancies. No other clinical information on these women are available to us. Some donors were also subsequently genotyped for a dimorphism at position 80 of HLA-C.
Recruitment	Women undergoing elective terminations were recruited. Women under the age of 18 are not recruited.
Ethics oversight	Ethical approval was granted by the Cambridge Research Ethics Committee (study 04/Q0108/23).

Note that full information on the approval of the study protocol must also be provided in the manuscript.

Flow Cytometry

Plots

Confirm that:

☒ The axis labels state the marker and fluorochrome used (e.g. CD4-FITC).

☒ The axis scales are clearly visible. Include numbers along axes only for bottom left plot of group (a 'group' is an analysis of identical markers).

☒ All plots are contour plots with outliers or pseudocolor plots.

☒ A numerical value for number of cells or percentage (with statistics) is provided.

Methodology

Sample preparation	Peripheral blood mononuclear cells (PBMCs) were isolated from whole blood by Pancoll (PAN-Biotech) and cryopreserved in 90% FCS/10%DMSO. To isolate decidual mononuclear cells (dMCs), decidual tissue pieces were first washed in RPMI-1640 and remaining blood clots and vessels removed using scalpels. Remaining tissue was then minced and 5ml of Collagenase IV (0.1g/100ml, sigma) in RPMI-1640 with 10% FCS added for further dissociation by GentleMACS. The tissue was incubated for another 45 minutes at 37oC whilst being gently shaken. The collagenase was quenched by addition of RPMI-1640 and then filtered through 100um and then 40um filters. dMCs were isolated following a Pancoll centrifugation step and then cryopreserved in 90%FCS/10%DMSO.
Instrument	FACS data was acquired on a BD LSR Fortessa (BD Biosciences). CyTOF data was acquired on an Helios (Fluidigm)
Software	FCS files were analysed with FlowJo v10.5.3 (Tree Star Inc.). tSNE, DensVM clustering and nearest neighbour-based residual probability analyses were performed using the R packages cytofkit (v.1.6.5) and DepecheR (v1.1.9) from Bioconductor (Chen et al., 2016; Theorell et al., 2019).
Cell population abundance	Subsets were excluded if they contained fewer than 100 cells. For confocal microscopy at least 10 000 cells were stained and subsequently analyzed, and representative cells were analyzed in detail for extraction of data as in figures.
Gating strategy	For CyTOF: Beads excluded using Ce140 channel, Singlets were gated upon using the parameter Event Length, Live cells were gated using Rhodium, CD45+ cells were selected, then CD3-CD14-CD19-HLA-DR- were identified. Further gating is described in text/figure legend. For flow cytometry and for FACS sorting prior to microscopy, live lineage negative cells expressing NK cell markers such as CD94/NKG2A or CD56. See methods and figure legends for detailes

☒ Tick this box to confirm that a figure exemplifying the gating strategy is provided in the Supplementary Information.
